# A Cross-Sectional Analysis of Dietary Intake and Nutritional Status of Patients on Haemodialysis Maintenance Therapy in a Country of Sub-Saharan Africa

**DOI:** 10.1155/2021/1826075

**Published:** 2021-05-15

**Authors:** Nyangi A. Gityamwi, Kathryn H. Hart, Barbara Engel

**Affiliations:** Faculty of Health and Medical Sciences, University of Surrey, Guildford, UK

## Abstract

Malnutrition is common among dialysis patients, but there is insufficient literature on the problem from resource-poor settings of the sub-Saharan region. We conducted a cross-sectional investigation of dietary intake and nutritional status of haemodialysis (HD) patients to inform the current status of this population group in the region. HD patients aged ≥18 years, with dialysis vintage of ≥3 months, at one nephrology unit in Tanzania were assessed for their habitual diet and nutrient intake. Anthropometric measures and biochemistry tests were also performed. The diet was predominantly starchy food based, accompanied by a limited selection of vegetables. Fruits and animal protein were also minimally consumed (1 portion/day each). Fruit consumption was higher in females than males (median (25^th^, 75^th^) = 2 (1, 2.3) versus 0.5 (0, 1.7) portions, *p* = 0.008). More than 70% of participants had suboptimal measures for protein and energy intake, dietary iron, serum albumin, muscle mass, and hand grip strength (HGS). Inadequacies in protein and energy intake and dialysis clearance (URR) increased with the increase in body weight/BMI and other specific components (MAMC and FMI). Consumption of red meats correlated significantly and positively with serum creatinine (*r* = 0.46, *p* = 0.01), potassium (*r* = 0.39, *p* = 0.03), and HGS (*r* = 0.43, *p* = 0.02) and was approaching significance for a correlation with serum iron (*r* = 0.32, *p* = 0.07). C-RP correlated negatively with albumin concentration (*r* = −0.32, *p* = 0.02), and participants with C-RP within acceptable ranges had significantly higher levels of haemoglobin (*p* = 0.03, effect size = −0.28). URR correlated negatively with haemoglobin concentration (*r* = −0.36, *p* = 0.02). Patients will benefit from improved nutritional services that deliver individually tailored and culturally practical dietary advice to enable them to make informed food choices whilst optimizing disease management.

## 1. Introduction

Dietary inadequacy and protein energy wasting (PEW) are not uncommon amongst patients with end-stage renal disease (ESRD). PEW affects 30–75% of patients [1] and is associated with adverse clinical outcomes, poor quality of life, and increased mortality [2].

Several nutritional practice guidelines are in place to help patients maintain optimal nutrition. Adult patients are recommended to have an adequate energy intake ranging from 30–40 kcal/kg/day [3, 4] to enable them preserve their protein stores throughout the disease progression [5, 6]. Similarly, adequate protein intake (≥1.1 g/kg of ideal body weight per day), at least 50% of which should be of high biological value (HBV), is also recommended for dialysis patients in order to adequately supply all essential amino acids [3, 4]. This is to account for the additional protein losses and inflammation-induced protein catabolism associated with dialysis [7]. The National Kidney Foundation (NKF) [8] quantifies this in practical terms as 6 to 11 servings of carbohydrate and three servings (a total of 8 to 10oz or 200 to 250 g) of HBV protein such as meat or fish per day. NKF also recommends the consumption of 2–3 portions of fruits and vegetables every day to meet patients' micronutrient needs. However, there is also a requirement to select carbohydrate and protein foods and fruits and vegetables with low phosphorus and potassium contents and a need to consider the ‘hidden' phosphate in food additives and preservatives [9–11].

Despite the presence of dietary guidelines, dialysis patients have generally lower dietary intakes than the healthy population [12], decreasing even further as their disease progresses [13]. This is commonly attributed to poor appetite or anorexia due to the buildup of uremic toxins [7, 14, 15]. The numerous dietary restrictions related to sodium, potassium, phosphorus, as previously mentioned, and also to fluid, further exacerbate poor intakes [11, 16]. Socioeconomic influences such as financial status may further influence the dietary choices and adequacy in haemodialysis patients. Patients living alone or on a low income may face difficulties preparing appropriate meals and adhering to the renal diet due to limited finances, poor physical strength, and lack of time (due to treatment). This can be a particular problem in developing countries, including sub-Saharan Africa (SSA), where there may be limited access to HBV protein sources which are also normally expensive [17]. Poor literacy and a shortage of renal dietitians/nutritionists pose further challenges for ensuring the adequacy of patients' dietary intakes in many countries in the SSA region. Even where dietitians are available, they are not enough of them [18] rendering nutrition care suboptimal in this region.

Regular assessment and monitoring of dietary intake and nutritional status will help to highlight areas of inadequacy for proper nutritional management of dialysis patients, but to the best of the author's knowledge, this is not routinely carried out for ESRD patients in this country and there are no published reports on this subject. The objectives of this study were to investigate and present the dietary intake and nutritional status of haemodialysis patients in a single but major dialysis clinic in the country to inform local community and healthcare providers and add to the scarcely available literature for this setting.

## 2. Materials and Methods

The study adopted a cross-sectional design and was conducted at the nephrology unit in Dar es Salaam, Tanzania. It included adult patients (aged ≥ 18 years) who had been receiving HD maintenance therapy for at least three months at the time of the study. Patients who were severely ill and those with communication difficulties were excluded. The consenting participants were interviewed for the collection of personal data including age and comorbidities. They also had their nutritional status and dietary intake assessed and blood tests performed.

### 2.1. Dietary Intake Assessment

Patients were interviewed to assess their habitual food intake using a food frequency questionnaire (FFQ) modified from Beck at al. [19]. Patients were asked how frequently they had consumed different foods over the previous one-month period with intake frequency rated as never eaten, eaten less than once/month, 1 to 3 times/month, once a week, 2 to 3 times/week, 4 to 6 times/week, once/day, 2 to 3 times/day, and 4+ times/day. Mean nutrient and energy intakes were estimated using the multiple-pass 24-hour recall method (MPR) [20] on three days of the week (dialysis day, weekend nondialysis, and weekday nondialysis day). A photographic Atlas of food portions from the Abu Dhabi Food Control Authority [21] was used to guide patients' estimations of their portion sizes (in grams). Dietary energy and protein content of all consumed foods were estimated for each participant, using a local food composition table compiled by Lukmanji et al. [22]. In order to compare food consumption with the NKF recommendations, the food amount consumed in grams (collected through MPR) was then converted into standard portion sizes using the conversion guide available within the NKF online learning resources [8], for example, ½ cup or 1 small fruit is equal to one serving/portion and a one serving of carbohydrate is equivalent to a ½ cup of cooked rice or 60gm. Nutrient intakes were adjusted for energy intake (1000 kcal) to allow comparison of nutrient density between genders in addition to absolute intakes.

### 2.2. Nutritional Assessment

Measures of nutritional assessment were dry body weight, height, mid-arm circumference (MAC), triceps skinfold thickness (TSF), hand grip strength (HGS), and fat mass index (TMI), lean mass index (LMI), and body mass index (BMI).

MAC was measured using a nonstretch measuring tape (®Seca). TSF was measured using the Harpenden skinfold calliper. Mid-arm muscle circumference (MAMC) was then calculated using the formula MAMC = MAC-TSF (all measured in cm).

Body height was measured using Seca 755 (USA) dial column weight scale with height rod attached without shoes and measured to the nearest 0.1 cm. For convenience, height measurements were taken before dialysis when patients were measuring their predialysis body weight.

LMI, FMI, body weight, and BMI data were estimated after dialysis using a portable Bioimpedance Analysis (BIA) machine (BF508, Omron Healthcare Co., Ltd., Kyoto, Japan).

HGS was measured before starting dialysis using a digital dynamometer (Takei 5401, Takei Scientific Co., Ltd., Japan). Subjects stood in a steady position holding a dynamometer with the nonfistula arm and pressed as hard as possible for 3 seconds with the arm was positioned at the side of the body. Three measurements were taken from each participant, and the mean was recorded.

### 2.3. Clinical Outcome Measures

The study utilised data on clinical outcome measures that were routinely collected by the clinics. Results of serum creatinine, urea, albumin, haemoglobin, ferritin, iron, potassium, phosphate, adjusted calcium, and C-reactive protein (C-RP) were retrieved from the clinical database. For this study, the most current monthly results (performed closest to the point of data collection) were used.

### 2.4. Statistical Analysis

All data were analysed using IBM SPSS (v27). Data were checked for normality and then descriptively summarised using the mean (SD) or median (25^th^, 75^th^ percentiles) as appropriate. All nutrient data were presented as the mean daily intakes derived from 3-day food recalls. Mann–Whitney *U* tests or independent *t* tests and chi-square test were used to compare the intakes between males and females, and correlation analysis was performed to study the association between variables of nutrient intake and indicators of nutritional status (e.g., albumin, muscle mass, fat mass, and lean mass). All statistical tests were two sided with a significance level of 0.05.

## 3. Results

### 3.1. Demographic, Anthropometric, and Biochemical Characteristics of the Study Participants

Of the 120 HD patients in the unit during the study period, 77 patients (64%) consented to take part in the study and male outnumbered female (57%, *n* = 44) participants.

Table 1 presents demographic, anthropometric, and biochemical data for all participants and by gender. The median (25^th^, 75^th^ percentiles) age was 49 (43, 56) years and dialysis vintage ranged from 3–32 months. The common reported comorbidities were hypertension (54%), diabetes (2%), and a combination of diabetes and hypertension (44%).

The Mann–Whitney test indicated no gender difference in dialysis vintage. Compared to female participants, males were older (ns); had significantly higher HGS (*p* < 0.001) and MAMC (*p* = 0.049); and had statistically higher BMI and LMI (both ns). Females had significant higher FMI (*p* = 0.008). Male participants also had significantly higher serum albumin (*p* = 0.002) and creatinine (*p* = 0.006) and lower urea reduction ratio (URR) than females (*p* = 0.009). There were no gender differences in serum C-RP, potassium, phosphorus, or calcium levels. Males had significantly higher ferritin levels, and although serum iron, transferrin saturation percentage (TSAT%), and haemoglobin concentration were higher, these differences were not significant (Table 1).

### 3.2. Description of Dietary Intake of the Study Participants

A total of 51 patients (66% of the sample) completed an interviewer-administered FFQ, and 42 (54.5%) participants completed a 3-day recall. Female participants were more likely to respond to both the FFQ and the recalls than males (FFQ: 82% v 54.5%, *x*^2^ = 5.11, *p* = 0.024; recalls: 76% ‘v' 39%, *x*^2^ = 7.93, *p* = 0.005).

#### 3.2.1. Frequency and Type of Protein and Energy Foods Consumed

The diet was predominantly starch-based (including cereals, grains, roots, and tubers), with these foods consumed most frequently as in Table 2.

**Figure 1:**
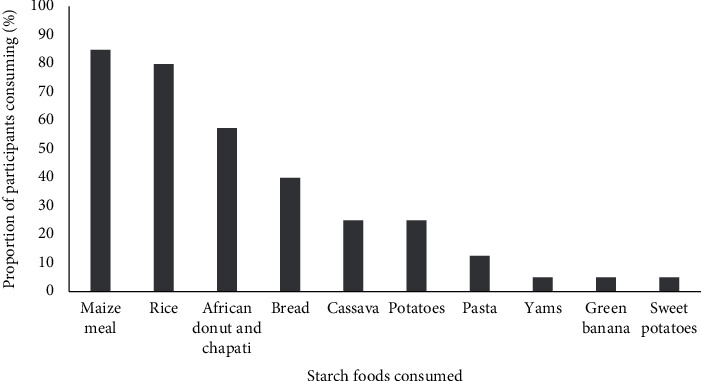
Different types of starch staple foods that were commonly consumed during the study period by adults on haemodialysis in one Tanzanian centre (*n* = 51). The bars represent the percentage of participants reporting consumption of each food type.

Rice and maize meal/stiff porridge (*Ugali*) were the most consumed starch dishes, reported to be consumed by 83% and 87% of the FFQ respondents, respectively. Conversely, cassava, potatoes, and green bananas were only reported to be consumed by less than 5% of the FFQ respondents (Figure 1).

**Figure 2:**
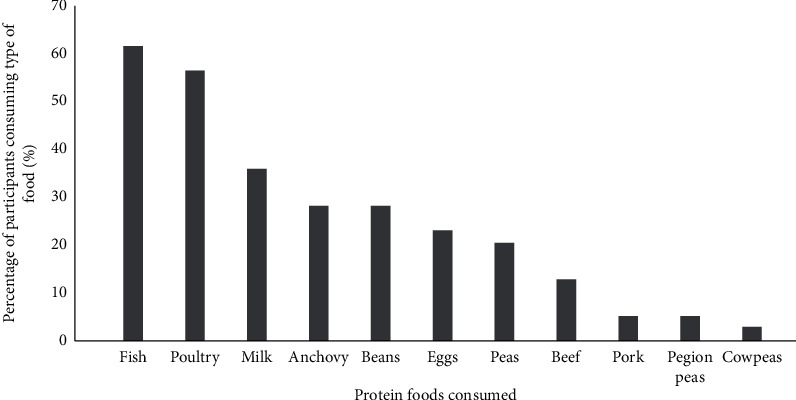
Different types of protein foods that were commonly consumed during the study period by adults on haemodialysis in one Tanzanian centre (*n* = 51). The bars indicate the percentage of participants reported consuming each type of food.

A low consumption frequency of animal protein was observed, and red meat (liver, beef, and pork) was rarely consumed during the study period. Fish was the most consumed animal protein food (Figure 2). However, the quantities/portions of fish consumed were very small, equivalent to 0.3 of the standard portion per day.

Analysed by gender, a significantly higher animal protein consumption (*p* = 0.04) and close-to-significantly higher starchy food consumption (*p* = 0.06) was seen in by males as compared to their female counterparts. However, this significance was not retained for either starches (*p* = 0.12) or protein (*p* = 0.85) when corrected energy intake (expressed per 1000 kcal).

#### 3.2.2. Frequency and Types of Fruits and Vegetables Consumed

**Figure 3:**
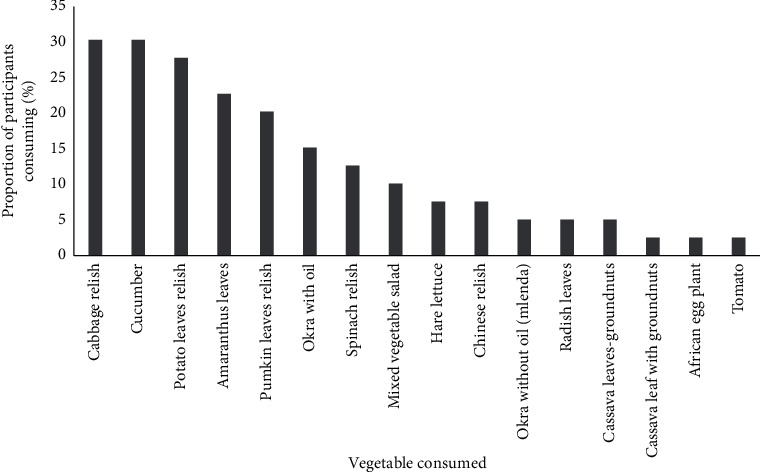
Different types of vegetables that were commonly consumed during the study period by adults on haemodialysis in one Tanzanian centre (*n* = 51). The bars indicate the percentage of participants reported consuming each type of vegetables.

**Figure 4:**
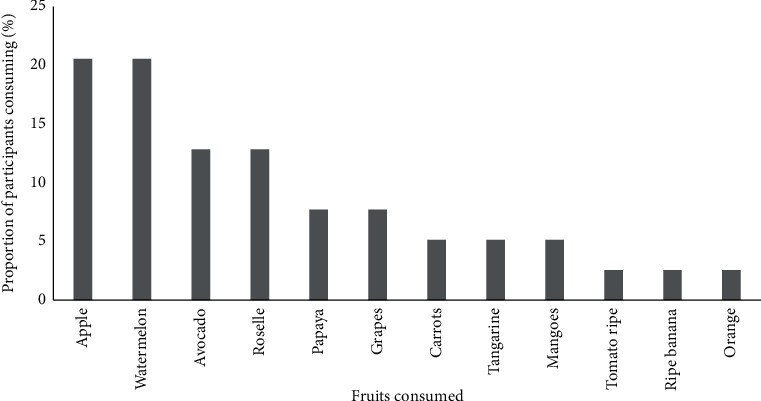
Different types of fruits that were commonly consumed during the study period by adults on haemodialysis in one Tanzanian centre (*n* = 51). The bars indicate percentage of participants reported consuming each type of fruits.

Participants reported vegetable consumption at least twice daily, whereas fruits were consumed on average once a day (Table 2). A narrow selection of fruits and vegetables was reported; vegetables were mainly limited to cucumbers and cabbage (Figure 3), whereas fruits were predominantly watermelon and apples (Figure 4).

Compared to males, female participants had significantly higher consumption of fruits even after correcting for energy intake (*p* = 0.02). There was no significant gender difference in vegetable intake (Table 2).

### 3.3. Nutrient Intake and Nutritional Status of Study Participants

When selected measures of dietary intake and nutritional markers were compared with the recommended reference levels from the European Best Practice Guideline (EBPG), the majority of participants had inadequate intakes of protein, energy, and dietary iron (85%, 75%, and 84%, respectively).

In terms of nutritional markers, the majority of participants had below normal HGS (73%), albumin levels (80%), and MAMC (80%) (see Table 3). Only for BMI did the majority of male participants meet the recommendations of being above the level of 23 kg/m^2^.

There was no gender difference in attainment of recommended protein and energy intakes, but a significantly higher proportion of male participants met their gender-specific recommended iron intake as compared to their female counterparts (35.3% ‘v' 4.0%, *p* = 0.01. see Table 3).

There was no gender difference in the percentage attaining the recommended level for BMI or gender-specific HGS. Males had significantly better attainment of albumin, but the majority had MAMC measures below the 50^th^ percentile of the 2003–2006 NHANES data (Table 3).

### 3.4. Association between Dietary Intake, Nutritional Status, and Clinical Outcome Measures

Spearman correlation analysis showed no association between demographic parameters of age or dialysis vintage and dietary intake of energy, protein, or iron (all *p* > 0.05). Similarly, age and dialysis vintage were not associated with the studied clinical outcome measures (URR, C-RP, albumin, potassium, calcium, phosphorus, or haemoglobin) or nutritional markers (BMI, muscle mass, fat mass, and lean mass). The exceptions were with creatinine (*r* = −0.38, *p* ≤ 0.01) and hand grip strength (*r* = −0.40, *p* ≤ 0.01), both of which decreased with an increase in participants' age.

Controlling for gender, protein and energy intake (corrected for body weight) were significantly and negatively associated with BMI, MAMC, and FMI (Table 4). There was a trend towards lower FMI among participants with protein intake above 1.1 g per kg of body weight per day. There was also a trend towards lower FMI and significantly lower BMI and MAMC at the higher levels of energy intake, i.e., above 30 kcal/kg/day (Table 4). Controlling for gender and age, there was no association between energy or protein intake with HGS.

There were no significant associations between protein and energy intake and any of the studied clinical outcome measures (Table 5). However, there was a positive and significant association between amount of red meat (beef, goat, lamb, and pork) consumed and predialysis serum creatinine (*r* = 0.46, *p* = 0.01), potassium (*r* = 0.39, *p* = 0.03), MAMC (*r* = 0.45, *p* = 0.02), and HGS (*r* = 0.43, *p* = 0.02) and a trend towards an association with serum iron concentration (*r* = 0.32, *p* = 0.07). There was also a significant positive association between the amount of starchy foods eaten and potassium levels (*r* = 0.39, *p* = 0.02).

URR correlated significantly and negatively with MAMC, creatinine, potassium, and haemoglobin. MAMC, LMI, and haemoglobin levels were significantly higher among those participants with a URR below 65% (Tables 4 and 5).

There was no direct association between C-RP and either of the stated nutritional indices or clinical measures, except for albumin which was significantly and negatively associated with C-RP (Tables 4 and 5). When grouped according to C-RP levels, lower haemoglobin levels were observed among participants with C-RP above 5 mg/l (Table 5).

## 4. Discussion

Chronic Kidney Disease (CKD) patients need to adhere to a range of dietary restrictions to reduce toxin accumulation resulting from their renal insufficiency. Unfortunately, this also makes them prone to dietary inadequacy and puts them at an increased risk of developing PEW. Regular optimal nutritional counselling and monitoring of patients' nutritional status could help ensure adequate nutrition for this population group and so improve their treatment outcome and reduce their mortality [27, 28]. This cross-sectional study aimed to provide a snap shot of dietary intake and nutritional status of patients receiving haemodialysis at one major clinic in Tanzania, a country in sub-Saharan Africa.

The median age of dialysis patients (49 years) in the present study was substantially lower than that reported in developed countries including UK, where the majority of patients are in their sixth to seventh decade [29]. However, the age was comparable with other dialysis populations in African countries such as Ghana (43.86 yrs) [30], Nigeria (42.55 yrs) [31], South Africa (43.4–55 yrs) [32], and Cameroon (47.4 yrs) [33]. There is a possible indication of earlier onset and/or progression of CKD to ESRD in the region, as suggested by other authors [34–36]. Lack of CKD awareness, delayed diagnosis, and management of CKD and/or aetiological chronic conditions such as diabetes [37] and hypertension [38, 39] play a significant role in the rapid progression of CKD to ESRD in developing countries. An earlier study conducted in Tanzania revealed that only 10% of individuals with CKD in a sample of 606 participants from 431 urban and rural households were aware of their condition [40]. This emphasizes the need for routine health checks specific to the population at risk of developing CKD as well as establishing appropriate management protocols for diabetes, hypertension, and CKD in order to delay progression.

The patients' diets were predominantly starch based, with maize meal and rice most frequently eaten, whereas starchy roots and tubers such as cassava, potatoes, and plantains reported to be minimally consumed. A similar pattern was observed in previous studies conducted in a cohort of patients with HIV [41] and within a general urban population [42] in the country. In all these studies, carbohydrate or starch-based food contributed the greatest proportion of energy intake. The Tanzanian food consumption data from the 2010/11 Tanzanian National Panel Survey (TZNPS) [43, 43] also reported starchy roots and cereals such as maize, rice, and cassava as the main staple foods consumed by the general population at the national level. As such, the dietary habits observed in this cohort of HD patients follow a pattern which is similar to the region with slight dietary restriction of foods known to be problematic for their potassium levels such as cassava and potatoes.

Generally, patients were estimated to consume just above 500 g of carbohydrate/starch food per day. This is equivalent to 2.5–3 cups of cooked rice or *Ugali* (the commonly eaten starches). Referring to the NKF recommendation which suggest an intake of 6 to 11 portions/servings per day (one serving of cooked rice = 1.5 cups) [8], the consumption in the present cohort was at the lower end of this recommendation (considering 3 cups of cooked rice/Ugali = 6 servings). Consequently, the inadequacy is translated to the total energy intake as, regardless of gender, the majority of participants did not meet the recommended daily energy intake of ≥30 kcal/kg/day [4]. This is of concern as inadequate energy may reduce the benefit of protein by catabolizing it for energy [45] and is independently associated with all-cause mortality [27].

Protein sources were mainly fish and chicken (reported by more than 50% of respondents) though consumed in small quantities. On average, they were consumed once a day, and the intake amount was estimated to be 113g per day, which can be translated to about two-thirds of a cooked chicken breast or less than a palm of cooked fish fillet (significantly lower than this among female participants). This is below the recommended intake of at least 3 portions/servings daily (a portion in every main meal) [8]; consequently, the majority of participants, irrespective of gender, did not meet their daily total protein intake recommendation of ≥1.1 g/kg body weight/day [4]. Nevertheless, daily consumption of protein and carbohydrate in this cohort was higher than that reported for the general population (237 g carbohydrate and 38 g total protein) and HIV-infected women (407 g carbohydrate and 76.1 g total protein) [41] in the country and a neighbouring country of Kenya (296–321 g carbohydrate, 43–48 g protein) [45]. Although the intake of this cohort seems to follow the common dietary pattern of the area, or even performed better, their intake does not meet the increased protein and energy requirements associated with their HD, and this is of concern.

The choice of fish and chicken was interesting considering that they are expensive compared to other animal protein sources such as beef, dairy, eggs, nuts, or legumes and are normally less frequently consumed in the general population [42]. At the time of this study, the price of beef steak was 10,000 tsh/kg (approximately 5USD or 4GBP) whereas chicken and fish were priced at equivalent to USD8 or 6GBP per kilo. Beans (the most common plant protein) cost approximately 2000 tsh/kilo (1USD or 0.8GBP). Anecdotal evidence from participants suggested that their choice of chicken and fish was driven by the common clinical advice due to concerns regarding phosphate content or urea accumulation from plant protein, dairy, and red meat, respectively. Participants admitted facing challenges in affording the fish and chicken, and this is probably why they were consumed in small quantities. Unfortunately, socioeconomic evaluation of participants was beyond the scope of this study; therefore, we could not ascertain their association with food habits. Nevertheless, the previous reports in the country suggest that income plays a positive role in dietary quality [41, 47].

Generally, there appeared to be a fundamental challenge of both inadequate total food intake and inadequate food quality. Patients' meals were monotonous and lacked dietary variety. With such inadequacy, it is unlikely that adults on HD will meet recommendations for other nutrients (e.g., micronutrients). This is further compounded by the low reported consumption of fruits and vegetables. The present cohort reported to consume on average only 1 portion of fruit and vegetables per day instead of 2–3 portions recommended by the NKF [8]. With concerns regarding the potassium content of fruits, patients reported having been advised to consume green apples and red grapes. Unfortunately, grapes are seasonal fruits and, therefore, not always available, and apples are expensive as they are mainly imported, rendering this advice impractical. However, a similar trend of low consumption of fruits and vegetables was observed in an in-country report of a general urban population where respondents consumed on average 0.2 and 0.7 servings of fruits and vegetables, respectively, in a 24-hour recall assessment [42]. This food culture may partly explain even lower levels of intake observed in the present cohort of dialysis patients who need to follow a potassium-restricted diet as part of their disease management. Low consumption of fruits and vegetables among dialysis patients was also evident elsewhere within the sub-Saharan setting [48], suggesting this to be a shared problem in the region.

Gender differences in food intake were evident; female intakes of fruits and vegetables exceeded those of male participants, whereas their carbohydrate and protein intakes were lower. Similar gender differences in protein and vegetable intakes, but not fruits and carbohydrate intake, were also reported in an HIV cohort investigated in the country [49]. Thus, the gender differences in dietary intake observed in the present cohort could be culture based, and it is suggested that women are more conscious about dietary choices and tend to eat home-made meals than males in the region. Nevertheless, it is of paramount importance that tailored nutrition education is provided to patients with renal failure which recognises their nutritional need, personal characteristics, and disease status. Practical advice encouraging a range of local and seasonal fruits and vegetables with acceptable potassium contents could help increase their intake. This study has highlighted a clear need to translate clinical guidelines into practical recommendations for patients which are cognisant of local food availability and population resources.

Nutritional status as denoted by BMI was at the borderline (23.1 kg/m^2^) of the optimal range which is not satisfactory as the levels are likely to fall below the recommendations. It should also be noted that BMI is not a sensitive marker of muscle mass. It is possible for patients to have muscle loss despite high or normal BMI because the body weight used in BMI calculation does not distinguish between muscle mass and body fat. Some studies have shown a low HGS in conjunction with an increase in body fat and body weight [49, 50]. Furthermore, a greater BMI has only been found to be protective in patients with normal or high muscle mass [51]; hence, a muscle function test such as HGS is normally performed and interpreted alongside BMI. The majority of participants had low muscle mass compared to the reference general population of the NHANES 2003–2006 population, and their hand grip strength could be categorised as being sarcopenic [23]. This has considerable implications for health outcomes and quality of life among dialysis patients [52–54]. Although the use of NHANES MAMC data and European definition of sarcopenia as a reference point for this African ethnic and, in particular, dialysis group could be questionable, the classifications derived from these references are supported by the dietary intake data previously reported. Nevertheless, BMI and MAMC observed in the present study are comparable to the haemodialysis patients from other countries of sub-Saharan Africa [48, 55], suggesting a common regional problem.

In the present study, protein and energy intakes (corrected for an individual's body weight) were significantly negatively associated with BMI, muscle mass, and fat mass index. This finding is contrary to the expectation that higher energy intake would be associated with higher BMI and muscle or fat mass but was confirmed (for energy) by analysis comparing the named nutritional markers between patients falling below versus within recommendations. This suggests that the attainment of recommended energy and protein intake decreased with the increase in some body composition parameters, highlighting that patients with bigger stature or more muscle mass are less likely to meet their protein and energy intake requirement. This again emphasizes the need for personalized nutritional care rather than the general daily dietary recommendations that do not take into consideration individual body requirements. It was also of concern that URR was negatively associated with muscle mass as this highlights that there could be an inadequate dialysis clearance among patients with higher muscle mass.

Although there was no association between protein or energy intake with either of the studied clinical outcome measures, the level of predialysis serum creatinine, potassium, and iron was positively associated with the amount of red meat (beef, lamb, goat, and pork) consumed. This is not an unusual finding as most animal protein foods are known to have high contents of iron and potassium and contribute to metabolic end products including creatinine. Whilst observational studies suggest beneficial effects, in terms of better survival among haemodialysis patients with high protein intake of above 1.1 g/kg/day [5, 56], the issue of parallel increases in phosphorus and potassium intakes and an increased risk of metabolic acidosis must be borne in mind, particularly where there is inadequate dialysis [10, 57]. Adoption of cooking techniques, including boiling and straining of cooking liquid, would also help reduce potassium content from foods.

The intake of red meat (beef, goat, lamb, and pork) was associated with serum iron but not haemoglobin concentration, a possible reflection of a compromised iron metabolism due to the uremic and inflammatory state of patients. Indeed, the median level of C-RP was above 5 mg/l, the safe level suggested in the literature [58–60], and there were significantly lower haemoglobin levels among participants with C-RP of above 5 mg/l compared to those with lower levels.

The findings from this study illustrate that good nutrition is still a challenge to dialysis patients in the region. Individual tailored and culturally practical dietary advice would support patients to choose an appropriate quantity and quality of food to optimise their management from the food available to them. Capacity building through training of local nutritionists and adaptation of the international nutritional guidelines into culturally appropriate and practical advice for patients could help improve nutritional care of renal patients and, hence, improve their treatment outcome and survival.

### 4.1. Study Limitations

This study is presented with several limitations. Because of the cross-sectional and observational nature of the present study, the cause-effect relationship between dietary intake and nutritional status or clinical outcome measures could not be substantiated. The sample size was small limiting data analysis (e.g., sub-group gender analyses) and reducing the power of the study. A multicentre study could have helped increase the size. Some important data were either missing or not available as part of routine clinical measures at the time of data collection, hence limiting further investigation of the dietary habits and/or nutritional status observed in the present cohort. Future studies should explore the possibility of collecting data on dialysis treatment (e.g., type of dialysate, vascular access, and Kt/v), additional clinical outcome measures (urea, prealbumin, and transferrin), and additional comorbidities (only diabetes and hypertension were available in the current study). The study also lacks socioeconomic data such as education level and employment/income status that would have helped to contextualise the food habits. Almost half of the male respondents did not answer the food frequency questionnaires, and therefore, the findings could be poorly representative of this gender. There were several limitations inherent in the estimation of individual dietary intakes due to the local food culture of preparing food without standard recipes and sharing a pot meal. Moreover, there are no established local food portion sizes and dietary guidelines for CKD; therefore, in the present study, estimations and references were based on UK and USA standards which might not be practical or appropriate in this local context.

## 5. Conclusions

The diets of adults on haemodialysis in this Tanzanian centre were monotonous with a narrow selection of food options. Females were more likely to consume fruit and vegetables whereas males were more likely to have higher intakes of energy and protein. Although there was no direct association between dietary intake and nutritional status, there is a suggestion that patients with greater muscle mass and weight were less likely to meet their recommended weight-based energy and protein intakes. There was also a suggestive positive advantage of red meat consumption on iron (but not haemoglobin) status and a proposed negative impact of inflammation and uremic state on their levels. There is a need for the nutritional service to be improved in the country, to enable adequate nutritional evaluation and provision of individualized, culturally tailored, and socioeconomically sensitive dietary guidance for the well-being of CKD patients and other population groups with special nutritional requirements. Further research is required to quantify the relative influence of socioeconomic factors (e.g., income and literacy level) and dialysis treatment on dietary intake and nutritional status of dialysis patients.

## Figures and Tables

**Table 1:** Demographic, anthropometric, and biochemical characteristics of adult haemodialysis patents in one Tanzanian centre.

	All (*n* = 77)	Male (*n* = 44)	Female (33)
Age (years)	49 (43, 56)	49.0 (43.0, 56.5)	45.5 (32.8, 59.3)
Dialysis vintage (months)	7 (3, 16)	7 (3, 11)	7 (3, 22)
BMI (kg/m^2^)	23.1 (20.4, 26.4)	23.9 (21.4, 25.9)	22.1 (19.1, 27.2)
MAMC (cm)	23.4 (21.1, 25.7)	24.5 (22.2, 26.3)^**a**^	22.4 (19.9, 24.9)^**b**^
FMI (kg/m^2^)	3.9 (1.8, 7.5)	2.4 (06, 6.1)^**a**^	5.1 (3.1, 11.7)^**b**^
LMI (kg/m^2^)	18.4 (16.3, 20.8)	19.1 (18.4, 22.3)	16.3 (15.3, 17.9)
HGS (kg)	21.1 (±9.3)	25.4 (±9.8)^**a**^	16.1 (±5.5)^**b**^
Serum albumin (g/l)	36 (34, 39)	38.5 (34.8, 40.0)^**a**^	34.00 (32.0, 37.0)^**b**^
Serum creatinine (*µ*mol/l)	812 (538, 1099)	968 (677, 1268)^**a**^	593 (393, 993)^**b**^
Urea reduction ratio	67 (56, 77)	59 (54, 73)^**a**^	75 (64, 83)^**b**^
C-RP (mg/l)	6.1 (3.2, 16.9)	6.1 (2.4, 14.7)	6.1 (4.2, 46.0)
Potassium (mmol/l)	5.1 (4.3, 6.3)	5.2 (4.4, 6.3)	5.1 (4.1, 6.5)
Phosphorus (mmol/l)	1.1 (0.9, 1.4)	1.2 (0.9, 1.5)	1.1 (0.8, 1.3)
Adj. calcium (mmol/l)	2.1 (2.0, 2.2)	2.1 (2.0, 2.2)	2.1 (2.0, 2.2)
Serum iron (*µ*mol/l)	9.0 (6.0, 12.5)	10.8 (7.7, 14.0)	7.0 (4.7, 10.1)
Ferritin (*µ*g/l)	151.2 (53.7, 283.6)	207.9 (101.4, 308.4)^**a**^	79.0 (38.0, 227.6)^**b**^
Transferrin saturation percentage (TSAT%)	17.6 (13.9, 21.0)	17.9 (15.7, 20.8)	15.5 (9.8, 25.0)
Haemoglobin (g/l)	87.1 (±16.7)	88.3 (±16.3)	85.5 (±17.2)

BMI = body mass index; C-RP = C-reactive protein; FTI = fat-tissue index; HGS = hand grip strength; LTI = lean-tissue index; MAMC = mid-arm muscle circumference; data are presented as median (25^th^, 75^th^ percentiles) or mean (±Standard deviation); ^(a,b)^significant difference between male and female participants, *p* = 0.05, Mann–Whitney test.

**Table 2:** The consumption frequency and estimated intake of selected foods for adults on haemodialysis in one Tanzanian centre, overall and by gender.

Food type/group	Consumption frequency (*n* = 51)	^†^Average daily intake (gm), median (25^th^, 75^th^ percentiles)		
		All (*n* = 42)	Male (*n* = 17)	Female (*n* = 25)
Meat, fish, and poultry and eggs (MFP)	Once/day	113 (50, 167)	166 (65, 196)^a^	71 (31, 155)^b^
Vegetables	2–3/day	76 (0, 156)	32 (0, 122)	79 (15, 172)
Fruits	4–6/week	1 portion (0, 2.5)	0.5 (0, 2)^a^	2 (1, 3)^b^
Starches	2–3/day	508 (359, 617)	544 (507, 690)^a^	440 (324, 560)^b^
Protein (g/kgwt/day)		0.6 (0.5, 0.9)	0.69 (0.6, 1.1)	0.53 (0.4, 0.9)
Energy (kcal/kgwt/day)		24.4(18.4, 30.8)	27.2 (22.9, 31.4)	21.8 (17.2, 30.8)
Total iron intake (mg/d)		7.2 (5.0, 9.7)	7.2 (5.4, 9.8)	6.3 (4.7, 9.2)

Starches = all cereals, grains, and tubers; MFP:1 portion = 90–140 g; vegetables: 1 portion = 1/2cup = 75 g; fruits: 1 portion = 1/2 cup = 1 small fruit; starches: 1 portion = 1/2 cup = 60 g. Data are expressed as median (25^th^, 75^th^). ^(a,b)^Cells with different superscript letters are significantly different between genders according to the Mann–Whitney test, *p* < 0.05. ^†^Assessed by the multiple-pass 24-hour recall method (MPR).

**Table 3:** The proportion of adults on haemodialysis from one Tanzanian centre (*n* = 77) with intakes of selected nutrients and markers of nutritional status within and outside of the recommended ranges, overall and by gender.

Parameter	Proportion of participants with measures within and out of recommended ranges, % (*n*)			Gender comparison (*P*, effect size)
	All	Male	Female	
Protein (g/kgwt/d) [4]				0.21, −0.22
≥1.1	15.4 (6)	25.0 (4)	8.8 (2)	
<1.1	84.6 (33)	75.0 (12)	91.3(21)	
Energy (kcal/kgwt/d) [4]				
≥ 0	25.6 (10)	25 (4)	26.1 (6)	1.0, 0.01
<30	74.4 (29)	75.0 (12)	73.9 (17)	
Total dietary iron (mg/day) [4]				
Male ≥ 8 mg	16.7 (7)	35.3 (6)	4.0 (1)	**0.01**, −0.41
Female ≥ 15 mg				
Male < 8 mg	83.3 (35)	64.7 (11)	96.0 (24)	
Female < 15 mg				
BMI (kg/m^2^) [4]				0.25, −0.18
≥23	50.8 (31)	59.4 (19)	41.4 (12)	
<23	49.2 (30)	40.6(13)	58.6 (17)	
Albumin (g/l) [4]				
≥40	20.0 (14)	31.6 (12)	6.3 (2)	**0.02**, −0.32
<40	80.0 (56)	68.4 (26)	93.8 (30)	
HGS (kg) [23]				
Male > 30	27.1 (16)	28.1 (9)	25.9 (7)	1.0, −0.03
Female > 20				
Male ≤ 30	72.9 (43)	71.9 (23)	74.1 (20)	
Female ≤ 20				
MAMC (cm) 50^th^ percentiles [24]				
Male ≥ 29.5	19.6 (11)	3.4 (1)	37.0 (10)	**0.005**, 0.42
Female ≥ 23.6				
Male < 29.5	80.4 (45)	96.6 (28)	63.0 (17)	
Female < 23.6				

BMI = body mass index; HGS = hand grip strength; MAMC = mid-arm muscle mass; [24] UK Renal Association; [25] National Kidney Foundation-Kidney Disease Outcome Quality Initiative; [23] 2003–2006 NHANES data; [4] European Best Practice Guideline; [22] European Working Group on Sarcopenia in Older People; ^⸸^chi-square test.

**Table 4:** The anthropometric markers (median) split by attainment of relevant reference ranges of dietary intakes (*n* = 42), dialysis adequacy (*n* = 50), and inflammation status (*n* = 62) for adults on haemodialysis in one Tanzanian centre.

	BMI (kg/m^2^)	HGS (kg)	MAMC (cm)	FMI (kg/m^2^)	LMI (kg/m^2^)
Protein (g/kgwt/d) [4]					
<1.1	22. 5 (20.4, 28.5)	18.7 (15.5, 25.0)	22.9 (20.9, 25.6)	5.7 (2.5, 11.7)	17.70 (15.45, 18.8)
≥1.1	20.9 (19.2, 24.1)	22.7 (16.9, 30.9)	21.6 (19.0, 24.8)	1.9 (1.6, 5.2)	17.50 (17.1, 19.8)
^#^(*p*, effect size)	0.17, −0.22	0.32, −0.17	0.22, −0.21	0.06, −0.29	0.41, −0.13
^¥^(*r*, *p*)	−0.47, <0.01	0.09, 0.62	−0.52, <0.01	−0.39, 0.02	−0.14, 0.41
Energy (kcal/kgwt/d) [4]					
<30	22.6 (21.1, 28.9)	18.3 (14.2, 25.0)	24.0 (21.3, 25.9)	6.4 (2.5, 12.3)	17.7 (15.9, 18.9)
≥30	20.9 (18.7, 24.1)	21.1 (19.1, 30.9)	21.4 (19.0, 23.5)	3.1 (1.7, 5.2)	17.4 (14.4, 18.8)
^#^(*p*, effect size)	0.04, −0.33	0.11, −0.27	0.02, −0.39	0.08, −0.28	0.60, −0.08
^¥^(*r*, *p*)	−0.47, <0.01	0.25, 0.15	−0.46, <0.01	−0.37, 0.02	−0.20, 0.23
URR (%) [25]					
≤65	23.8 (21.0, 26.6)	21.5 (12.8, 29.9)	25.2 (23.1, 28.0)	3.5 (1.2, 7.6)	18.8 (17.9, 22.9)
>65	22.1 (19.5, 26.2)	18.6 (15.3, 24.2)	22.3 (20.6, 24.4)	4.3 (1.9, 11.2)	17.1 (15.3, 18.5)
^#^(*p*, effect size)	0.45, −0.11	0.46, −0.11	0.01, −0.45	0.44, −0.12	0.01, −0.38
^¥^(*r*, *p*)	−0.18, 0.25	0.04, 0.81	−0.43, <0.01	−0.15, 0.33	−0.10, 0.54
C-RP (mg/l) [26]					
<5	23.4 (20.5, 26.4)	20.1 (16.8, 28.2)	23.4 (22.0, 25.6)	4.1 (1.9, 9.4)	18.0 (15.5, 19.5)
≥5	22.6 (20.5, 26.2)	19.1 (13.7, 26.1)	23.7 (20.4, 25.6)	2.8 (0, 6.4)	18.8 (16.9, 22.0)
^#^(*p*, effect size)	0.89, −0.02	0.37, −0.12	0.56, −0.08	0.26, −0.15	0.32, −0.13
^¥^(*r*, *p*)	0.06, 0.68	−0.02, 0.89	0.03, 0.83	−0.03, 0.81	0.13, 0.33

URR = urea reduction ration; C-RP = C-reactive protein; [24] UK Renal Association; [25] National Kidney Foundation-Kidney Disease Outcome Quality Initiative; ^#^Mann–Whitney test; ^¥^covariate correlation coefficient.

**Table 5:** The clinical outcome measures (median) split by attainment of relevant reference ranges of dietary intakes (*n* = 42), dialysis adequacy (*n* = 50), and inflammation status (*n* = 62) for adults on haemodialysis in one Tanzanian centre.

	Albumin (g/l)	Creatinine (mmol/l)	Potassium (mmol/l)	Calcium (mmol/l)	Phosphate (mmol/l)	Haemoglobin (g/l)	Serum iron (*µ*mol/l)
Protein (g/kgwt/d) [4]							
<1.1	36.0 (34.0, 40.0)	859.2 (546.5, 1121.1)	5.1 (4.0, 6.3)	2.1 (2.0, 2.2)	1.1 (0.9, 1.4)	84.8 (78.0, 98.5)	9.4 (6.7, 12.0)
≥1.1	39.0 (33.8, 41.0)	780.2 (344.8, 1276.8)	4.4 (3.7, 5.2)	2.1 (1.9, 2.3)	1.1 (0.8, 1.1)	83.5 (59.1, 107.3)	8.8 (6.9, 15.0)
#(*p*, effect size)	0.45, −0.13	0.74, −0.05	0.27, −0.18	0.95, −0.01	0.32, 0.16	0.73, −0.06	0.95, −0.01
¥(*r*, *p*)	−0.13, 0.47	−0.11, 0.53	−0.05, 0.76	−0.02, 0.92	−0.12, 0.51	−0.19, 0.28	0.03, 0.85
Energy (kcal/kgwt/d) [4]							
<30	38.0 (34.5, 40.0)	851.1 (545.1, 1121.1)	4.9 (4.0, 6.3)	2.1 (2.0, 2.2)	1.1 (0.9, 1.4)	86.6 (80.5, 99.1)	9.4 (6.7, 11.7)
≥30	34.0 (33.0, 39.0)	913.4 (616.4, 1276.8)	4.95 (4.4, 5.7)	2.1 (1.9, 2.2)	1.1 (0.9, 1.3)	79.9 (66.9, 88.7)	8.8 (4.7, 15.2)
#(*p*, effect size)	0.13, −0.26	0.61, −0.08	0.89, −0.02	0.32, −0.16	0.91, −0.02	0.07, −0.30	0.93, −0.01
¥(*r*, *p*)	−0.16, 0.35	0.04, 0.81	0.14, 0.43	−0.16, 0.34	−0.04, 0.84	−0.12, 0.52	0.25, 0.14
URR (%) [25]							
≤65	37.5 (34.0, 39.25)	898.15 (554.0, 1346.48)	5.55 (4.63, 6.70)	2.06 (1.96, 2.17)	1.14 (0.79, 1.37)	90.55 (81.35, 104.75)	7.9 (6.0, 1.8)
>65	36.0 (32.0, 39.0)	855.2 (501.1, 1094.8)	5.1 (4.3, 6.1)	2.1 (2.0, 2.2)	1.0 (0.9, 1.5)	81.3 (70.6, 96.1)	9.1 (6.5, 10.9)
#(*p*, effect size)	0.49, −0.21	0.29, −0.13	0.22, −0.13	0.72, −0.07	0.83, −0.08	0.05, −0.28	0.96, −0.15
¥(*r*, *p*)	−0.19, 0.20	−0.33, 0.02	−0.29, 0.04	−0.07, 0.62	−0.13, 0.39	−0.36, 0.02	−0.09, 0.57
C-RP (mg/l) [26]							
<5	38.0 (35.0, 39.0)	927.6 (578.7, 1085.1)	4.9 (4.2, 5.9)	2.1 (2.0, 2.2)	1.2 (0.9, 1.5)	89.2 (84.7, 100.0)	9.2 (7.2, 12.5)
≥5	36.0 (32.0, 39.0)	687.8 (482.3, 1179.4)	5.1 (4.4, 6.7)	2.1 (1.9, 2.2)	1.1 (0.8, 1.4)	81.4 (70.5, 99.9)	8.3 (5.2, 12.5)
#(*p*, effect size)	0.09, −0.21	0.32, −0.13	0.29, −0.13	0.60, −0.07	0.55, −0.08	0.03, −0.28	0.24, −0.15
¥(*r*, *p*)	−0.32, 0.02	−0.08, 0.55	−0.02, 0.90	−0.09, 0.49	−0.09, 0.50	−0.22, 0.11	−0.07, 0.59

URR = urea reduction ration; C-RP = C-reactive protein; [24] UK Renal Association; [25] National Kidney Foundation-Kidney Disease Outcome Quality Initiative; ^#^Mann–Whitney test; ^¥^covariate correlation coefficient.

## Data Availability

The data used in the present study are not publicly available but can be made available from the corresponding author upon reasonable request.
